# A pipeline for rapid, high-throughput imaging and quantitative analysis of human intestinal organoids

**DOI:** 10.1371/journal.pone.0332418

**Published:** 2025-10-08

**Authors:** Faith M. Sawyer, Fabio Stossi, Erika Nachman, Robert A. Britton, Michael J. Bolt, Michael A. Mancini, Mary K. Estes, Sarah E. Blutt

**Affiliations:** 1 Department of Molecular Virology and Microbiology, Baylor College of Medicine, Houston, Texas, United States of America; 2 Integrated Microscopy Core, Baylor College of Medicine, Houston, Texas, United States of America; 3 Department of Molecular and Cellular Biology, Baylor College of Medicine, Houston, Texas, United States of America; 4 Alkek Center for Metagenomics and Microbiome Research, Baylor College of Medicine, Houston, Texas, United States of America; 5 Dan L. Duncan Comprehensive Cancer Research Center, Baylor College of Medicine, Houston, Texas, United States of America; 6 Department of Medicine, Baylor College of Medicine, Houston, Texas, United States of America; Università degli Studi della Campania, ITALY

## Abstract

Human intestinal organoids (HIOs) are a model system for studying human intestinal epithelium. Utilizing HIOs for high-throughput studies remains inefficient, as analyzing their cellular composition and responses to varying experimental conditions requires extensive time and labor. We describe a 96-well plate-based automated pipeline for rapidly imaging and quantifying fluorescent labeling in HIOs using a high-throughput confocal microscope and image analysis software. The pipeline was leveraged to quantify varying levels of cell proliferation among donor HIO lines in response to microbial products. Cytoplasmic fluorescence via antibody labeling was also quantified with the pipeline, enabling measurement of the prevalence of specific cell types in HIOs. This platform offers a novel approach to efficiently and rapidly image and quantify fluorescent staining and immunolabeling in HIOs and has many potential applications, including drug screening, toxicity testing, intestinal barrier integrity and transport studies, microbiome and host-pathogen interaction studies, and lineage tracking.

## Introduction

Human intestinal organoids (HIOs) provide a model to study human intestinal epithelial biology and intestinal disease pathophysiology. Tissue stem cell-derived HIOs capitalize on the inherent self-renewal and differentiation potential of intestinal stem cells by self-organizing into three-dimensional (3D) crypt-villus structures with proliferative, crypt-like domains and differentiated, villus-like domains, controlled by growth factors, that mimic the native intestinal architecture [1,2]. Among their many benefits for studying human intestinal biology, HIOs retain the genetic signatures of the donor, recapitulate intestinal disease phenotypes, and preserve intestinal segment-specific characteristics, thereby providing an *in vitro* model of many of the native intestine’s *in vivo* features [3,4]. HIOs have been used for screening therapeutics, studying the microbiome and its interactions with the epithelium, modeling intestinal diseases such as colorectal cancer and bacterial and viral infections [5–8], and more recently, for phenotypic analysis in high-throughput applications. HIOs can be grown as a two-dimensional (2D) monolayer on 96-well plates [9], where they lack a 3D structure but offer better scalability and reproducibility for high-throughput studies.

Recent studies have demonstrated that high-throughput confocal microscopy and automated analysis techniques can effectively evaluate 3D organoid morphological and structural features in healthy HIOs [10] and genomic and proteomic characteristics in cancer-derived intestinal organoids [10,11]. These approaches illustrate the potential of high-throughput confocal imaging and computerized analysis methods for phenotypic profiling in complex 3D organoid models. However, using 3D HIOs for high-throughput, physiologically relevant studies poses limitations and challenges compared to 2D cultures. While 3D HIOs enable the study of tissue patterning and villus-crypt microdomains, their 3D structure increases imaging complexity, as multiple optical sections must be acquired while imaging to capture the full structure, thereby increasing acquisition time and requiring greater processing power. As 3D HIOs vary in size, shape, and organization, performing high-throughput phenotypic analysis in different disease states using 3D HIOs is more difficult, as disease-related alterations in structure [12] may serve as a barrier in applying the same analysis method to all samples. Additionally, 3D HIOs provide limited physiological relevance for microbiome studies, as the apical surface of the epithelium is not exposed, and any microbial products would be applied to the basolateral surface. Together, these limitations point to 2D HIOs as a physiologically relevant, scalable system for high-throughput phenotypic studies, as they are more homogeneous across samples, enable faster imaging and analysis, and provide greater relevance for microbiome studies as the apical surface is exposed. Despite the advantages of using 2D HIOs to study the human intestinal epithelium, conventional methods of imaging and analyzing HIOs remain inefficient and time-consuming, slowing down research progress and serving as a barrier to implementing HIOs in high-throughput experiments and functional assays. Conventional methods for analyzing HIO phenotypes include immunostaining and confocal microscopy, flow cytometry, and transcriptional profiling, all of which are usually performed manually, are low-throughput, or require labor-intensive analyses. To develop a high-throughput analysis pipeline to rapidly profile HIO phenotypes, we automated image acquisition and analysis using a high-throughput, spinning disk confocal microscope, image postprocessing, and free, open-source software. This pipeline allows users to efficiently characterize HIOs at the cellular or molecular level in 96-well plates. We illustrate how the platform can quantify varying levels of proliferation among multiple donor HIO lines by quantifying nuclear fluorescence following treatment with bacterial products and demonstrate how it can be used for antibody staining quantification by measuring cytoplasmic fluorescence of a cell identity marker. As proof-of-concept, results from the pipeline showed strong correlation when compared to manual imaging and the traditional method of quantitation by flow cytometry. These applications demonstrate the pipeline’s capacity to detect inter-donor variability and illustrate its ability to detect cell-specific responses, highlighting the application of the pipeline for therapeutic screening and other high-throughput experiments.

## Materials and methods

### Establishment of HIO cultures

J1 (J1005), J2 (J1006), and J3 (J1014) were generated as previously described [2,7,13] at the Texas Medical Center Digestive Diseases Center Gastrointestinal Experimental Model Systems (GEMS) Core. The tet*NGN3*-HIE line was established as previously described [14].

### 2D monolayer plating

The outermost wells of a 96-well plate (Corning, 3595) were filled with 100 μL sterile DI water to maintain humidity of the plate. A stock solution of 1 mg/mL collagen IV (Sigma, C5533) in 100 mM acetic acid was diluted 1:30 in sterile DI water. 100 μL of diluted collagen solution was added to each inner well of the 96-well plate, incubated for 90 minutes at 37°C, then removed, leaving a collagen coating. Matrigel (Corning, 356231) containing 3D HIOs cultured for 5–7 days in L-WRN conditioned medium (described below) was collected by washing wells of a 24-well plate with 500 μL per well of an ice-cold solution of 0.5M EDTA in 1 × PBS (1:1000). Matrigel containing the HIOs was pelleted for 5 minutes at 400 × *g* at 4°C, the supernatant removed, and the Matrigel-HIO pellet resuspended in 500 μL 0.05% trypsin/0.5 mM EDTA that was incubated for 5 minutes at 37°C. The trypsin was then inactivated with 500 μL complete medium without growth factors (CMGF-: 500 mL advanced DMEM/F-12 [Gibco], 5 mL 100 × GlutaMax™ [Gibco], 5 mL 1M HEPES) containing 10% FBS. The HIOs were vigorously pipetted to disassociate them, passed through a 40-μm cell strainer (VWR 76327–098) to create a single-cell suspension, then centrifuged for 5 minutes at 400 × *g*. The supernatants were removed, and cells were resuspended in 200 μL L-WRN conditioned medium [9]: advanced DMEM-F12 (Gibco), 1 × GlutaMax™ (Gibco), 10 mM HEPES (Invitrogen), 50% WNT3A-conditioned medium produced from CRL-3276 cells (ATCC), 1 × B-27 supplement (Gibco), 1 × N-2 supplement (Gibco), 10 mM nicotinamide (Sigma, N0636) diluted in DI water, 500 μM N-Acetyl-L-cysteine (Sigma, A8199) diluted in DI water, 500 nM A 83–01 (Tocris, 2939) diluted in DMSO, 10 μM SB202190 (Sigma, S7067) diluted in DMSO, 50 ng/mL EGF (Invitrogen, PMG8043) diluted in 1 × PBS, 10 nM gastrin I (Sigma, G9145) diluted in 1 × PBS, and 10 μM Y-27632 (StemCell Technologies) diluted in 1 × PBS. Cells were counted using an automated cell counter (Bio-Rad, TC20), and seeded at the desired density in each collagen-coated well of the 96-well plate with 100 μL of L-WRN conditioned medium per well.

### Microbial supernatant generation

Two microbial strains isolated from the human intestine were identified by full-length 16S rRNA sequencing: *Ligilactobacillus salivarius* 112 (LS112) and *Lacticaseibacillus rhamnosus* 105 (LR105). 10 mL MRS broth (Fisher, DF0881-17-5) was inoculated with LS112 or LR105 from glycerol stock and grown overnight at 37°C. The MRS bacterial culture was sub-cultured at an OD_600_ of 0.1 in 5 mL differentiation medium (L-WRN conditioned medium as described above without WNT3A-conditioned medium, nicotinamide, SB202190, and Y-27632). The differentiation medium subculture was grown until the OD_600_ measured 0.5–0.6, then centrifuged at 3,000 rpm for 15 minutes and the pellet discarded. Supernatants were pH-neutralized with 10-M NaOH to a pH of 7.0–7.4, then filter-sterilized using a 0.22-μm filter and aliquoted in 100-μL aliquots into sterile PCR tubes and frozen at –20°C. control differentiation medium was also filter-sterilized, aliquoted, and frozen.

### Microbial supernatant EdU assay

The morning after monolayer plating, L-WRN conditioned medium was removed and 100 μL of prewarmed microbial supernatant or 100 μL differentiation control medium was added to the well. The following morning, 1 μL of a 10-mM EdU-DMSO (ThermoFisher, C10337) stock solution diluted 1:10 in CMGF- (described above) was added to each well. Following 24 hours of incorporation, monolayers were fixed and permeabilized, with the following steps performed for 30 minutes each using 100 μL of solution per well on a rocker at RT: 4% paraformaldehyde (PFA) diluted in 1 × PBS, 50-mM NH_4_Cl, and 0.1% Triton-X diluted in 1 × PBS. EdU staining was then performed using the ThermoFisher Click-iT™ EdU Imaging Kit (C10337) according to the manufacturer’s protocol, then monolayers were incubated in DAPI for 20 minutes to stain cell nuclei.

### Doxycycline induction of tet*NGN3*-HIEs and antibody staining

Monolayers of tet*NGN3*-HIEs [14] were seeded with 2 × 10^5^ cells per well. 2 days after plating, differentiation medium (described above) was added to the control wells and 0.5 μg/mL doxycycline in differentiation medium was added to the other wells to induce *NGN3* expression. Monolayers were cultured for 7 days, with the medium changed every other day. On the 7^th^ day, monolayers were fixed, quenched, and permeabilized, with the following steps performed for 30 minutes each using 100 μL of solution per well on a rocker at RT: 4% PFA diluted in 1 × PBS, 50-mM NH_4_Cl, and 0.1% Triton-X100 diluted in 1 × PBS. Monolayers were blocked using 100 μL 3% BSA in 1 × PBS for 15 minutes, then 100 μL of chromagranin A antibody (Immunostar, 20085) diluted 1:500 in 3% BSA in 1 × PBS was added and incubated overnight at 4°C. Monolayers were then washed with 3% BSA in 1 × PBS for 15 minutes on a rocker, incubated with anti-rabbit 488 antibody (Rockland, 611-141-122) diluted 1:1000 in 3% BSA in 1 × PBS for 1 hour at room temperature, protected from light, washed with 3% BSA in 1 × PBS for 15 minutes on a rocker, and then incubated with DAPI for 20 minutes to stain cell nuclei.

### Flow cytometric quantification of EdU uptake and CgA expression

For detection of EdU via flow cytometry, HIO monolayers were treated with microbial supernatants and controls and pulsed with EdU as described above. Following 24 hours of EdU incorporation, a single cell suspension was generated by gently washing HIO monolayers with 100 μL of prewarmed CMGF- to remove dead cells, then incubating them in prewarmed Accutase™ (StemCell Technologies) for 10 minutes at 37°C. Cells were detached by pipetting and 3 monolayers per donor per condition were combined into one flow tube with 400 μL CMGF- with 10% FBS. Flow tubes were centrifuged for 5 minutes at 400 × *g*, then washed with 1 mL 1% BSA in 1 × PBS. Cells were then fixed, permeabilized, and stained using the ThermoFisher Click-iT™ EdU Flow Cytometry Kit (C10633) following the manufacturer’s protocol. EdU-positive cells were quantified using an LSRII flow cytometer (BD Biosciences) at the Baylor College of Medicine Cytometry and Cell Sorting Core, and the single-cell population was gated using doublet discrimination, from which 10,000 events were analyzed. For quantification of CgA expression, tet*NGN3*-HIEs were plated and cultured with or without doxycycline for 7 days as described above. 3 monolayers per condition with two technical replicates each were used to create single cell suspensions and washed with BSA as described above, then fixed with 4% PFA for 45 minutes on ice. Following fixation, samples were washed with 2 mL cold PBST (0.1% Tween-20 in 1 × PBS) for 10 minutes, then centrifuged for 5 minutes at 400 × *g,* and blocked with 500 μL 5% donkey serum (Sigma Aldrich) in 1% BSA for 20 minutes on ice. Samples were then incubated in 350 μL of the primary antibody (Immunostar, 20085) diluted 1:2,000 in 1% BSA overnight at 4°C. Samples were washed with 1% BSA three times, then incubated with 350 μL anti-rabbit 488 secondary antibody (Thermo Fisher A21206) diluted 1:5,000 in 1% BSA overnight at 4°C. Samples were washed with 1% BSA three times and then resuspended in 1% BSA. CgA expression was quantified as described previously for EdU uptake quantification.

### High-throughput spinning-disk confocal imaging and postprocessing

HIO monolayers plated on 96-well plates (Costar, 3595) were imaged on a Yokogawa CV8000 high-throughput spinning-disk confocal microscope, located in the Baylor College of Medicine Integrated Microscopy Core, with sequential imaging of the fluorescent channels using a 4 × objective. DAPI was excited with a 405-nm laser, and EdU and the anti-chromagranin A antibody with a 488-nm laser. For each image and channel, 3 z-stacks were captured at 20 μm apart to account for plate unevenness. 9 images (fields of view, FOVs, overlapping 230 pixels to facilitate stitching) were obtained per well. Images were first postprocessed using the Yokogawa acquisition software, which applied automatic corrections for dark-frame subtraction and flat-field correction, geometric distortion, spectral unmixing, and channel alignment. 16-bit TIFF images were uploaded in Yokogawa CellPathfinder software, aligned, and max intensity projected to create whole-well images. For visualization purposes, the brightness and contrast were adjusted for the images, with the same correction applied to all 2D HIOs from the same plate, however, the quantification was performed on the unmodified images.

### CellProfiler pipeline for image analysis

Reconstructed whole well images for each channel were uploaded in CellProfiler (version 4.2.5) and a central crop area was created using the “Crop” module for each individual fluorescent channel with the following parameters: cropping shape = rectangle; cropping method = coordinates; cropping pattern = every; left and right rectangle positions = 1500, 4000, absolute; top and bottom rectangle positions = 1500, 4000, absolute. Pixels were quantified from each fluorescent channel using the “IdentifyPrimaryObjects” module with the following parameters: typical diameter of objects in pixel units = 2, 40; discard objects outside the diameter range = yes; discard objects touching the border of the image = yes, thresholding method = global. The rest of the parameters must be optimized depending on the experiment. For each object, the “MeasureImageAreaOccupied” module is used to quantify the total area that a fluorophore occupies in the whole image. Results were exported to a Microsoft Excel spreadsheet and the following formula applied to normalize the cell densities: (number of green pixels ÷ number of blue pixels) × 100. For anti-CgA fluorescent analysis, the above parameters were followed, but the following modification was applied: “IdentifyPrimaryObjects” was applied to the 488 channel images with the typical diameter of objects in pixel units = 10, 40, threshold strategy = adaptive, thresholding method = robust background with default threshold levels applied.

### Statistical analysis

All experiments were performed at least 3 times, with 3 technical replicates per donor (n = 3 donor jejunal lines) per condition. For flow cytometry, 2 technical replicates were used for each condition. All statistical analysis was performed in GraphPad Prism (version 10). Comparison between the mean of experimental and control groups for experiments with 3 technical replicates were performed using one-way ANOVA and Dunnett’s method for multiple comparisons. For flow cytometry analysis with 2 technical replicates, Student’s *T-*test was used to compare experimental and control groups. For all statistical tests, **p* *< 0.05 was considered statistically significant.

## Results

### Optimization of plating density

**Fig 1:**
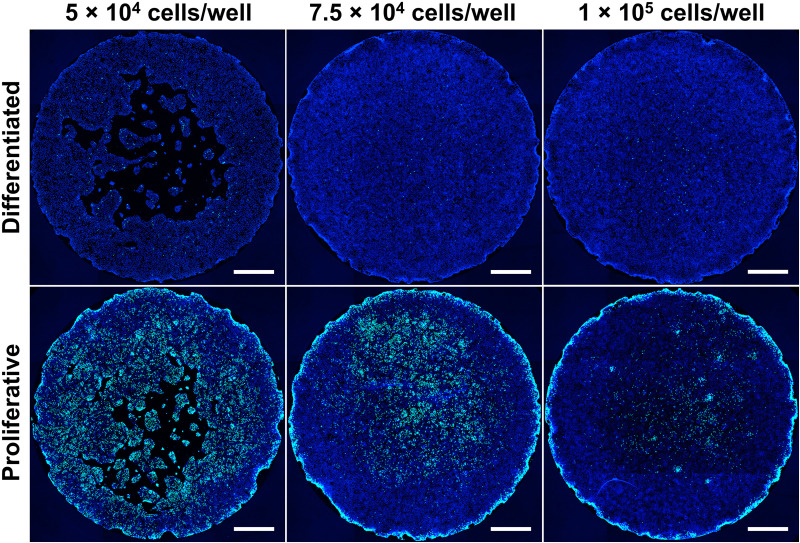
Effects of monolayer plating density on cell proliferation. Reconstructed whole well images of monolayers from a jejunal HIO line plated with varying cell densities and cultured for two days in differentiation medium or proliferation medium (L-WRN conditioned medium). HIOs were then pulsed with EdU for 24 hours and EdU-stained to label proliferating cells (green), then stained with DAPI (blue) to label remaining nuclei. Images were taken at 4 × magnification (scale bar = 1000 μm).

Using 2D HIOs as a model system for high-throughput studies requires optimization of cell-seeding density and confluency in the 96-well plate format. HIOs were seeded at densities ranging from 3.5 × 10^4^ to 1.4 × 10^5^ cells per well and cultured in L-WRN conditioned medium to induce a crypt-like (proliferative) state or differentiation medium to induce a villus-like (differentiated) state for one day after seeding. Following one day of culture in L-WRN or differentiation medium, 5-ethynyl-2’-deoxyuridine (EdU) was incorporated for 24 hours to label proliferating cells, and EdU uptake was detected using a click chemistry reaction that incorporates an Alexa Fluor 488 fluorescent tag. Whole well imaging of the 2D HIOs was performed using a Yokogawa CV8000 spinning-disk confocal microscope at 4 × magnification in two fluorescence channels: 405 nm for DAPI (to label cell nuclei) and 488 nm for EdU incorporation (to label proliferating cells) (Fig 1). It should be noted that the CV8000 is equipped with four laser lines, enabling additional multiplexing capabilities when required. Although the initial number of seeded cells necessary to reach the ideal confluency to detect proliferation varied by HIO line, the optimal seeding density allowed formation of an intact monolayer without covering the entire well, providing the cells with room to proliferate in the empty space over the period of time studied. For most HIO lines, this was between 5 × 10^4^ to 6 × 10^4^ cells per well. For the best results, the cell seeding density used to generate 2D monolayers in 96-well plates should be optimized for each donor HIO line prior to high-throughput microscopy studies.

### Whole-well imaging and postprocessing

**Fig 2:**
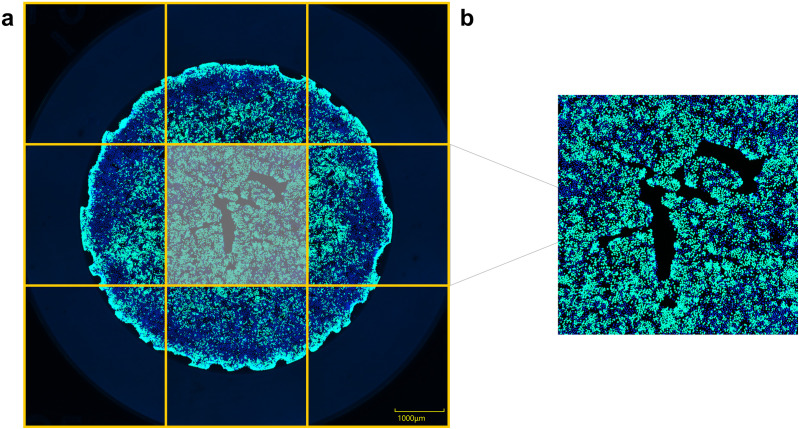
CV8000 image reconstruction and postprocessing. A) The whole well of an HIO monolayer was imaged on a CV8000 confocal by obtaining nine images of the well at 4 × magnification. Images were postprocessed and partial-well images were stitched together in CellPathfinder to reconstruct the whole-well image. B) To avoid edge effects, a central crop area was obtained from each reconstructed whole-well image by using the CellProfiler crop function before quantification.

Imaging HIOs remains a labor-intensive aspect of characterization, as it is traditionally performed manually via fluorescence microscopy. To accelerate and streamline the fluorescence microscopy imaging workflow, a high-throughput Yokogawa CV8000 spinning-disk confocal microscope was used to image the fluorescent signal in 2D HIOs. A benefit of using an automated, spinning-disk confocal microscope like the CV8000 compared to manual imaging methods is that after initially setting up the imaging parameters, all subsequent imaging is fully automated and very fast (approximately 1.5 minutes per well for two fluorophores). In contrast to standard confocal microscopes, which only provide a partial image of the well—potentially missing subtle phenotypic changes that are better observed when viewing the entire well—the CV8000 enables whole well imaging by capturing nine separate images per well (Fig 2A) that are then stitched together in postprocessing using Yokogawa’s CellPathfinder software. This postprocessing software also corrects for shading issues (i.e., uneven well surface or uneven illumination) and channel registration.

To extract quantitative information from each well, an image analysis pipeline was developed using the open-source software CellProfiler. Fluorescent staining at the edge of the well was found to be higher than the rest of the well (Fig 2A), which would artifactually skew the analysis of EdU-positive cells and total cell counts, therefore, a standard central cropped area was generated from each reconstructed whole-well image (Fig 2B) and pixel analysis was performed only on this central crop area to avoid edge effects skewing the quantification results. Blue and green pixels were quantified in the central crop area of each well using the CellProfiler program, with size criteria exclusions applied for the objects that were included in quantification to omit background signal and staining artifacts. To adjust for variable numbers of cells in each well and areas of the well that were empty due to the confluency level of the monolayer, a calculation to normalize the pixel quantification values according to the cell density was performed using the total area stained per well.

### Quantification of nuclear fluorescence using the HIO imaging and quantification pipeline

**Fig 3:**
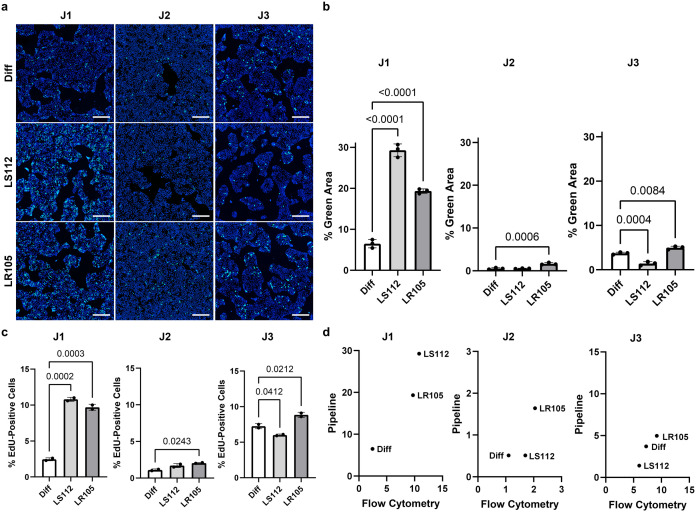
Pipeline identification of varying donor epithelial responses to microbial supernatants. HIO monolayers from three infant jejunal lines were treated with cell-free supernatants from *Ligilactobacillus salivarius* 112 (LS112) or *Lacticaseibacillus rhamnosus* 105 (LR105) grown in differentiation medium (Diff). Control monolayers were treated with Diff. Monolayers were pulsed with EdU for 24 hours. **A)** Monolayers were stained for EdU (green) to label proliferating cells and DAPI (blue) for cell nuclei. Images were captured using a CV8000 confocal at 4 × magnification (scale bar = 500 μm). **B)** EdU and DAPI-stained areas of each well were quantified via pipeline analysis of pixel staining and normalized for cell density. The percentage of green area was compared between supernatant-treated and Diff-treated wells using a one-way ANOVA. **C)** EdU-positive cells were quantified by flow cytometry. The percentage of EdU-positive cells was compared between supernatant-treated and Diff-treated wells using a one-way ANOVA. **D)** Correlation plots of pipeline-quantified percentage green area values averaged across the technical replicates plotted against the averaged flow cytometry EdU-positive values.

To evaluate the utility of the pipeline in addressing biological questions, the pipeline was used to screen bacterial secreted products for factors that promote HIO proliferation, using EdU incorporation as the readout. The high-throughput capability of the pipeline was leveraged to assess proliferation levels across five pediatric and five adult HIO lines. Supernatants from bacterial strains were collected at the logarithmic growth phase, filter-sterilized, and then applied to density-optimized HIO monolayers for 48 hours ahead of EdU and DAPI staining. Two human-isolated bacterial strains, *Ligilactobacillus salivarius* 112 (LS112) and *Lacticaseibacillus rhamnosus* 105 (LR105), were associated with varying levels of increased EdU fluorescent signal in the donor HIO lines (Fig 3) as quantified by both traditional fluorescent imaging (Fig 3A) and the automated pipeline (Fig 3B). On visual fluorescent microscopy inspection, qualitatively, the results of the monolayer staining (Figs 3A and S1) matched the quantification results obtained using the pipeline (Fig 3B) in both the central crop area of the images (Fig 3A) and the whole-well images (S1 Fig). The fluorescence quantification per well of HIO measured via pipeline was compared to quantitation obtained via flow cytometry (Fig 3C), a traditional approach to quantifying fluorescence in HIOs. The flow cytometry quantification confirmed the pipeline quantification results by showing consistency in the bacterial product that induced proliferation in each HIO donor line. When compared by correlation analysis (Fig 3D), the pipeline quantification and the flow cytometry quantification were highly correlated, with a Spearman’s rank correlation coefficient of **r* *= 1.0000, 0.8660, and 1.0000, for J1, J2, and J3 HIO lines, respectively. The agreement between the pipeline and flow cytometry quantification validates the capability of the pipeline to detect a wide range of donor-specific responses in HIOs and provides a foundation for its application as a tool for measuring variability in responses among multiple biological replicates.

### Quantification of cytoplasmic fluorescence using the pipeline

**Fig 4:**
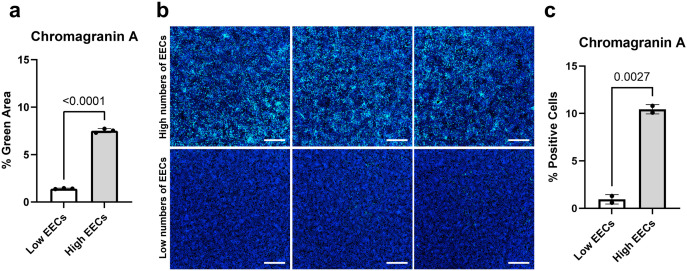
Chromagranin A staining in a neurogenin-3 inducible HIO line. Three monolayers of an inducible neurogenin-3 jejunal HIO line were cultured with differentiation medium. Three wells were induced with doxycycline to increase enteroendocrine cells (EECs) and three were not induced. **A)** Chromagranin A-stained and DAPI-stained areas of each well were quantified via pipeline analysis of pixel staining. The percentage green area was compared between low EEC (non-induced) and high EEC (induced) wells using an unpaired Student’s *t-*test. **B)** Monolayers were stained for chromagranin A (green) for EECs and DAPI (blue) for cell nuclei. Monolayers were imaged on a CV8000 confocal at 4 × magnification (scale bar = 500 μm). **c**, Chromagranin A-positive cells were quantified by flow cytometry. The percentage of Chromagranin A-positive cells was compared between low EEC (non-induced) and high EEC (induced) wells using an unpaired Student’s *t-*test.

To expand the capability of the pipeline to other experimental scenarios, the platform was used to quantify cytoplasmic fluorescence via the detection of fluorescently labeled antibodies to identify specific cell types. Enteroendocrine cells (EECs) in the intestinal epithelium contain cytoplasmic secretory granules filled with neuroendocrine factors. Under homeostatic conditions, EECs are rare in the intestinal epithelium, both *in vivo* and in HIOs [14,15]. To determine whether cytoplasmic immunolabeling of chromogranin A (CgA)—a well-characterized, EEC-produced factor—could be quantified using the pipeline, a genetically modified HIO line that can be induced with doxycycline to increase the numbers of EECs [14] was compared against the non-induced HIO line, which contains few EECs, using fluorescent antibodies against CgA. Immunolabeling with the CgA antibody resulted in nonspecific background cytoplasmic fluorescence, which led to artificially high pipeline quantification values for the wells not containing substantial numbers of EECs. To address this discrepancy, the pipeline’s thresholding strategy was adjusted in CellProfiler to remove background before quantification, which provided an accurate quantification of the central crop area by excluding nonspecific staining. After adjustment of the thresholding strategy, the pipeline quantification results (Fig 4A) showed that induced HIOs had a significant increase (*p* < 0.0001) in CgA immunolabeling compared to the non-induced HIOs, confirming the increased numbers of EECs in the induced wells. The pipeline quantification results corresponded to the visual increase in CgA fluorescent signal observed qualitatively using fluorescent microscopy (Figs 4B and S2), and quantitatively using flow cytometry (Fig 4C). As with the nuclear fluorescent quantification, visually comparing the reconstructed whole-well images (S2 Fig) to the pipeline quantification results served as a qualitative method to validate the quantitative pipeline data.

## Discussion

Here we described a rapid, automated, pipeline for imaging and quantifying nuclear and cytoplasmic fluorescence in HIOs. This method integrates whole-well confocal imaging with open-source image analysis and cell density-based normalization to enable scalable, unbiased quantification of fluorescence across large samples sizes. This pipeline supports analysis of both nuclear fluorescence and cytoplasmic antibody-based labeling, as demonstrated via experiments identifying donor-specific epithelial responses to identical microbial treatments and quantifying the prevalence of specific cell types within HIOs. These applications were validated qualitatively using manual immunofluorescence microscopy and quantitatively using flow cytometry, confirming their reliability compared to traditional methods of analysis. Although the platform was optimized for HIOs, this approach can be applied to any adherent cell model system that is amenable to the 96-well plate format and fluorescent labeling.

Fluorophore-based immunolabeling of intracellular components or structures is a common technique for identifying the presence and prevalence of a biological target within a population of cells. However, quantification of fluorophore labeling in HIOs via traditional methods like flow cytometry or manual confocal microscopy presents limitations. Flow cytometry requires cell disassociation, thereby removing spatial context and decreasing cell viability, and is poorly suited to high-throughput analysis due to the processing time needed for each sample. Manual imaging via confocal microscopy is labor-intensive, unsuitable for high-throughput experiments due to imaging time required, and prone to user bias as it relies primarily on qualitative assessment of the data and manual quantitation. In contrast, our pipeline combines open-source tools with a standardized imaging and quantification workflow, enabling unbiased image analysis and facilitating high-throughput experiments without substantially increasing labor and sample processing time. Furthermore, while many newer methods of HIO phenotypic analysis require advanced artificial intelligence or machine learning-based methods of analysis, this pipeline uses free, open-source software for analysis without the need for advanced algorithms or specialized training.

Key innovations of this platform include the use of whole-well imaging to provide a more comprehensive and unbiased representation of the data, a defined central crop area for analysis to standardize quantification, and normalization of fluorescence to cell density, which enables the study of HIOs under various experimental conditions. These features collectively minimize human bias and improve the rigor and reproducibility of image-based data while increasing the speed and ease of data acquisition and analysis.

While our pipeline streamlines analysis, its implementation depends on equipment and software availability. Although our pipeline is adaptable to images obtained via traditional, manual confocal microscopy, its high-throughput capacity is maximized with the use of a spinning-disk confocal microscope or other high-throughput microscopes. Implementation also requires optimization of the image analysis pipeline that depends on the complexity of fluorescent staining patterns and the levels of background fluorescence. Visual comparison of whole-well images to the pipeline quantification results serves as an internal qualitative validation step that strengthens scientific rigor and improves the overall reliability of the data by ensuring that quantification values are not skewed by unexpected fluorescent patterns, fluorescently labeled debris or other artifacts, or operator bias in setting threshold values. Here we have validated the pipeline’s application in quantifying two fluorophores, but this platform is compatible with multi-channel imaging of multiple fluorophores depending on the number of available laser lines on the microscope. However, when incorporating multiple fluorophores, there are several technical limitations that need to be considered. These include overlapping excitation and emission spectra of the fluorophores that may hinder accurate quantification, antibody sources and potential cross-reactivity (particularly when using multiple primary antibodies from the same species), limited availability and reduced signal of directly conjugated antibodies despite their ability to circumvent cross-reactivity, and the increased risk of autofluorescence with the addition of more fluorophores. Additionally, we find that strong fluorophores can overwhelm weaker signals, making simultaneous visualization of three fluorophores difficult without careful adjustment of gain and laser intensity.

This high-throughput imaging and quantification platform performs automated analyses of intracellular fluorescent signals in a heterogenous cell population, while enhancing accuracy in data analyses and allowing the cellular response in many biological replicates to be interrogated under the same conditions. Whereas here we showed a specific application of the pipeline in widely used HIO models that bridge the gap between *in vivo* animal studies and human clinical research, it is versatile and adaptable to other cell culture systems and organoid models. This pipeline effectively identifies varying donor responses under the same experimental conditions, highlighting its value for high-throughput studies that quantify the presence or absence of biological targets in response to therapeutics and probiotics, genetic modifications (e.g., CRISPR), and treatments like radiation or dextran sodium sulfate (DSS). Moreover, its application in clinical diagnostics holds the potential to revolutionize disease identification based on specific biomarkers that are retained in models that can be adapted to 96-well plates, like HIOs. By combining high-throughput capabilities with automated quantification, our pipeline offers a transformative approach to data analysis by drastically improving the efficiency and quality of image analysis and providing a more objective, scalable approach to quantifying biological markers. Due to its versatility in applications, this pipeline is poised to drive advancements in our understanding of gastrointestinal health and disease.

## Supporting information

S1 Fig Whole well images showing varying donor responses to microbial supernatants. HIO monolayers from three infant jejunal lines were treated with cell-free supernatants from *Ligilactobacillus salivarius* 112 (LS112) or *Lacticaseibacillus rhamnosus* 105 (LR105) grown in differentiation medium (Diff). Control monolayers were treated with Diff. Monolayers were pulsed with EdU for 24 hours and stained for EdU (green) and DAPI (blue). Images were captured using a CV8000 confocal at 4 × magnification (scale bar = 1000 μm).
(TIF)

S2 Fig Whole well images showing chromagranin A staining in a neurogenin-3 inducible HIO line. Monolayers of an inducible neurogenin-3 jejunal HIO line were cultured with differentiation medium. Three wells were induced with doxycycline to increase enteroendocrine cells (EECs) and three were not induced. Monolayers were stained for chromagranin A (green) for EECs and DAPI (blue) for cell nuclei. Monolayers were imaged on a CV8000 confocal at 4 × magnification (scale bar = 1000 μm).
(TIF)

S1 File Complete raw values used to generate all plots and run all statistical tests. Worksheets are organized by figure and retain the exact pairing/matching used in analyses.
(XLSX)
