# Supplementary figures and images for: A pipeline for rapid, high-throughput imaging and quantitative analysis of human intestinal organoids
Source: PLoS One. 2025 Oct 8;20(10):e0332418. doi: 10.1371/journal.pone.0332418 (PMC12507275; doi:10.1371/journal.pone.0332418)

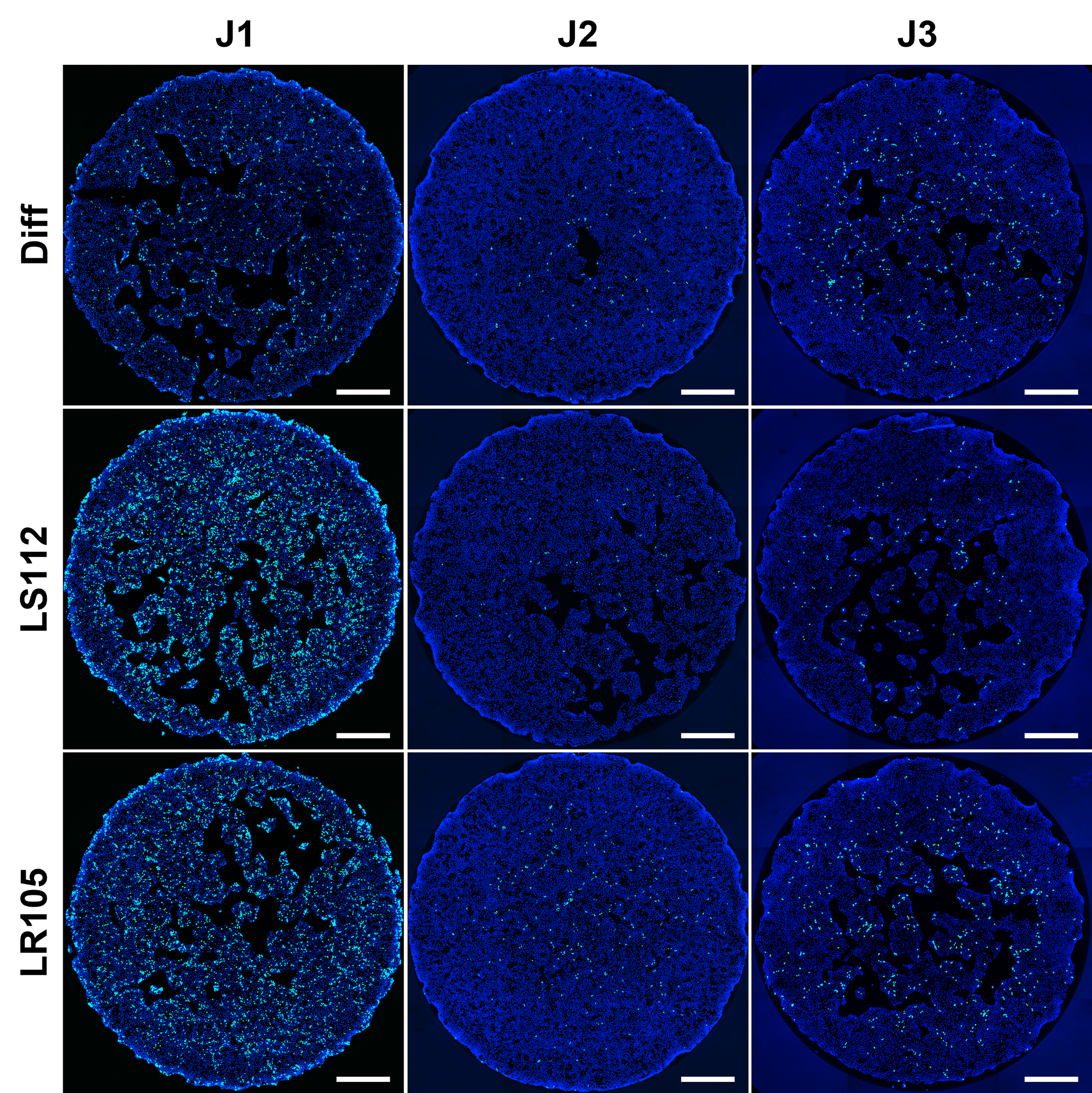

Supplement: S1 Fig — HIO monolayers from three infant jejunal lines were treated with cell-free supernatants from Ligilactobacillus salivarius 112 (LS112) or Lacticaseibacillus rhamnosus 105 (LR105) grown in differentiation medium (Diff). Control monolayers were treated with Diff. Monolayers were pulsed with EdU for 24 hours and stained for EdU (green) and DAPI (blue). Images were captured using a CV8000 confocal at 4 × magnification (scale bar = 1000 μm). (TIF) [file pone.0332418.s001.tif]

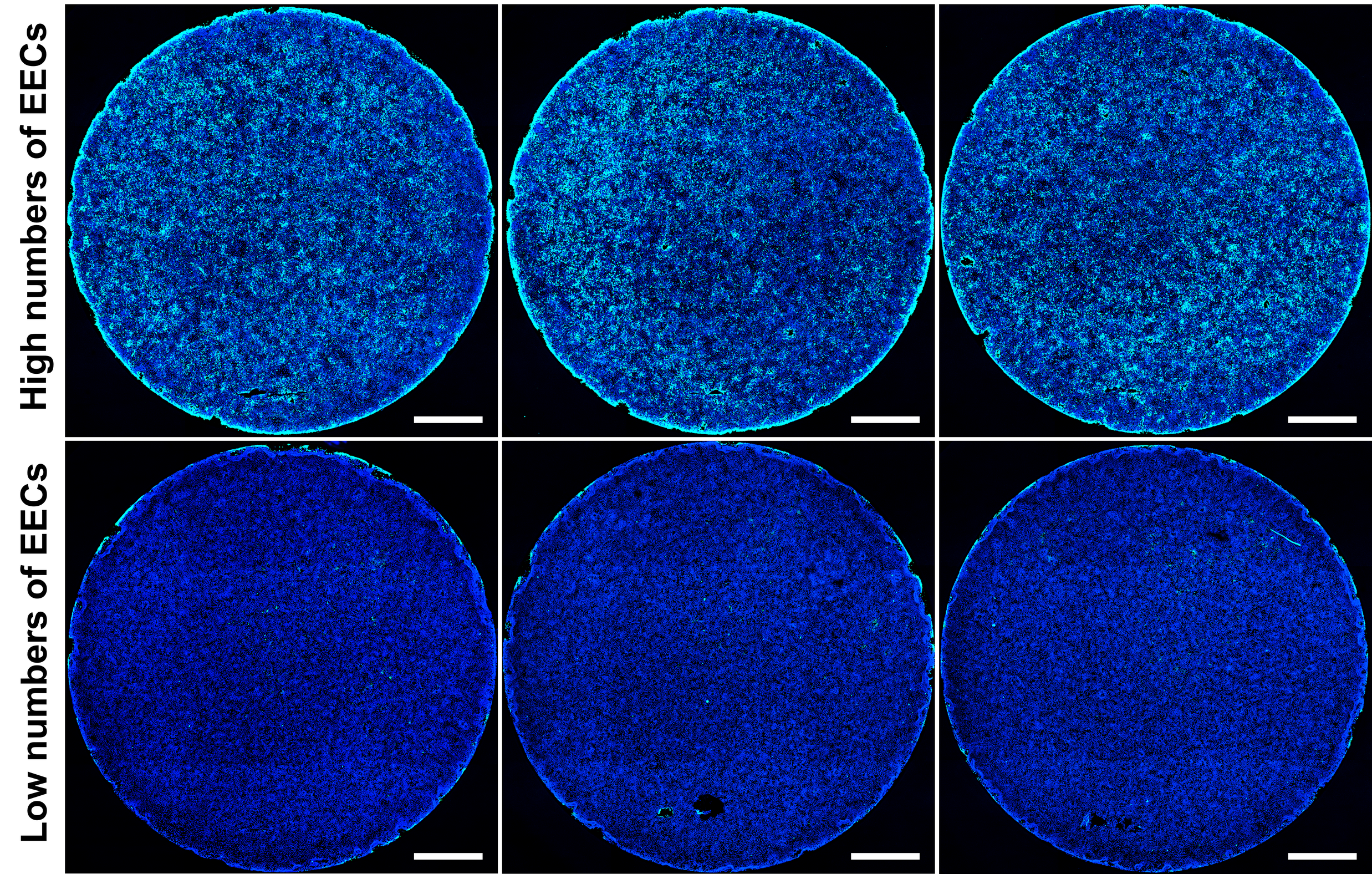

Supplement: S2 Fig — Monolayers of an inducible neurogenin-3 jejunal HIO line were cultured with differentiation medium. Three wells were induced with doxycycline to increase enteroendocrine cells (EECs) and three were not induced. Monolayers were stained for chromagranin A (green) for EECs and DAPI (blue) for cell nuclei. Monolayers were imaged on a CV8000 confocal at 4 × magnification (scale bar = 1000 μm). (TIF) [file pone.0332418.s002.tif]
